# Postpartum Depression and Maternal-Infant Bonding Experiences in Social Media Videos: Qualitative Content Analysis

**DOI:** 10.2196/59125

**Published:** 2025-05-15

**Authors:** Kunmi Sobowale, Jamie Sarah Castleman, Sophia Yingruo Zhao

**Affiliations:** 1 Department of Psychiatry and Biobehavioral Sciences, University of California Los Angeles, 760 Westwood Plaza, Los Angeles, CA, 90024, United States, 1 3107947035; 2 Ponce Health Sciences University School of Medicine, St. Louis, MO, United States; 3 University of California Los Angeles, Los Angeles, CA, United States

**Keywords:** postpartum depression, perinatal depression, postpartum anxiety, depression, TikTok, social media, video, content, postpartum, anxiety, maternal-infant, qualitative, content analysis, bonding, experience, deductive, inductive, regression model

## Abstract

**Background:**

While the negative effects of postpartum depression on maternal-infant bonding are well-documented, our understanding of how it exerts these effects remains incomplete. A better understanding of how maternal postpartum depression affects bonding can enable clinicians to better identify and support mothers with difficulties bonding with their children.

**Objective:**

This study aims to describe the bonding experiences of mothers with postpartum depression through an analysis of short-form videos and user engagement.

**Methods:**

We collected publicly available highly-viewed TikTok videos using hashtags associated with postpartum depression and associated engagement metrics in May 2023. After manual screening, we extracted 533 videos related to the mother-infant bond, from which we analyzed a random subset of 159 videos. We abstracted categories from videos using a hybrid deductive and inductive approach. Negative binomial regression models of video likes, views, shares, and comment count were used with content categories and the creator’s numbers of followers as independent variables.

**Results:**

Abstraction of content from mother-infant bond videos resulted in six categories: (1) navigating anxiety and anger, (2) creating physical and emotional boundaries, (3) overwhelmed by demands of caregiving, (4) subverted expectations, (5) enduring and finding strength through the challenge of postpartum depression, and (6) can’t remember early life. Subverted expectations and navigating anxiety and anger categories were associated with increased views (rate ratio [RR] 1.72, 95% CI 1.22‐2.43; RR 1.61, 95% CI 1.09‐2.38, respectively), likes (RR 3.61, 95% CI 2.55‐5.11; RR 3.96, 95% CI 2.69‐5.85, respectively), shares (RR 2.95, 95%CI 2.09‐4.18; RR 2.45, 95% CI 1.66‐3.61, respectively), and comments (RR 2.78, 95% CI 1.97‐3.94; RR 1.89, 95% CI 1.28‐2.79, respectively). Sensitivity analysis with creators with fewer followers mostly aligned with these results.

**Conclusions:**

This qualitative content analysis of short-form videos identified specific ways postpartum depression impacts the mother-infant bond, highlighting strategies for clinicians to support bonding. Analysis of engagement metrics further demonstrated the types of experiences that most resonate with viewers. Our findings demonstrate the potential of this qualitative method to augment understanding of lived experiences.

## Introduction

A mother’s bond to her child in the first few years of life greatly influences the child’s development and future mental health. The bond is a caregiver’s emotions, cognitions (ie, thoughts and beliefs), and behaviors toward the infant [1,2]. Bonding promotes child development, including physical, cognitive, and socioemotional domains [3-5], with more potent effects for the latter postnatally [3-7]. Longitudinally, stronger postpartum bonding is associated with less child internalizing and externalizing problems [6-8].

While the negative effects of poor maternal-infant bonding (MIB) are well-documented, our understanding of bonding, particularly in the context of depression, is lacking. Postpartum depression afflicts at least 13% of all birthing mothers annually in the United States and can harm child development and mental health in part by impairing mother-child bonding [7,9]. Yet, recent studies on the operationalization of the MIB find the construct, content, and structural validity of MIB measures, including measures validated in women with postpartum depression, to be insufficient [10,11]. A lack of diverse samples in measurement development may contribute to this issue [11]. Most qualitative studies focus on maternal depression more broadly rather than the MIB in mothers with depression. Such studies could provide valuable insights about the MIB in the context of postpartum depression [12]. A better understanding of how postpartum depression exerts this effect can help clinicians identify and support mothers with difficulties bonding with their children.

One way to increase understanding of MIB is to examine the experiences of women with postpartum depression, as described on social media. With the rise of social media, mothers have taken to the internet to discuss mental health difficulties using a variety of platform types (blogs, microblogs, forums, photo sharing, etc). Women share experiences of their own accord, allowing for the concerns of those with lived experiences of postpartum depression to come to light. Understanding these experiences is crucial as social approval, fear of judgment, or fear of having their child removed may make women less likely to disclose their concerns to clinicians [13]. Existing studies primarily use text-based social media such as forums [14-18]. To our knowledge, no studies to date have used video-based social media to examine MIB in the context of postpartum depression. Video-based social media data can provide a detailed understanding of the experiences of women with postpartum depression as compared to text alone, capturing nonverbal communication and additional contextual factors. TikTok, a social media platform featuring short-form videos, has significantly higher use (over 1 billion monthly active users) [19] than text-based platforms used in prior studies, potentially allowing for more diverse perspectives and broader reach.

Therefore, this study aims to categorize common ways mothers endorsing postpartum depression describe their bond with their child using content analysis of highly accessed videos on TikTok. In addition, this research examines user engagement (ie, views, likes, comments, and shares) across these categories.

## Methods

### Overview

We conducted a qualitative content analysis of videos from TikTok to document the variety of experiences of bonding in mothers with postpartum depression. Our approach was hybrid in nature, combining data-driven coding with literature-based coding [20]. The study followed the Standards for Reporting Qualitative Research reporting guideline [21].

### Ethical Considerations

The University of California, Los Angeles Institutional Review Board (#23-001157) deemed this nonhuman participant research exempt. Although we used publicly available data, given reidentification risks, we deidentified data throughout the study by not using identifiable information. Furthermore, we do not provide hashtags used for this study.

### Data Collection and Preprocessing

We identified videos using hashtags associated with postpartum depression, a common method in social media studies [22]. Content creators’ use of these hashtags suggests that they may self-identify with this diagnosis or its symptoms. Specific hashtags were identified using a variety of sources. First, the term “postpartum depression” was searched using the TikTok search feature. Common hashtags used in the initial 20 videos were collected. In addition, hashtags for postpartum depression that were used in other social media studies were evaluated [15]. We chose the 3 hashtags with the most views. Using these hashtags, we used the app Apify in May 2023 to collect publicly available popular videos and associated engagement metrics. These hashtags had a combined 1.1 billion views when videos were collected in May 2023.

We manually reviewed videos to exclude videos without content related to postpartum depression, and subsequently, not related to the mother-infant bond. We also excluded videos that were not in English, although the speakers were not required to be native English speakers. Given the large number of relevant videos (n=533) and resource constraints, we decided to use a random sample of approximately a third of the total number of videos collected (159/533, 30%) for analysis. This number of videos is in line with other content analysis studies of TikTok, which examine between 25 and 150 videos [23-26].

### Engagement Metrics

To explore user engagement with videos, we examined video views, likes, shares, and comments. Views indicate how many people were exposed to the content. The number of likes shows how many people responded positively to a video by endorsing it. Shares capture the number of times users spread the video to their network, indicating how much it spread beyond the original audience. A high share count suggests that a video garnered enough interest to be actively passed on. The number of comments reflects how much discussion the video generates by prompting reactions and opinions from viewers.

### Content Analysis

The research team consisted of a male psychiatrist with experience working with mothers with postpartum depression and infant mental health, along with a female medical student and a female undergraduate student who are interested in obstetrics and child development and psychology, respectively (KS, JSC, and SYZ).

A coding rubric was developed in several stages using an inductive and deductive (ie, hybrid) approach [27,28]. Videos were the unit of analysis with visual, audio, and text (both in the video and its description) components. First, deductively, one author (KS) identified an initial set of codes based on existing literature to design a preliminary codebook. Subsequently, over 5 rounds, coders independently watched 45 videos (5‐10 per round) from the eligible videos and then reviewed them as a group. In initial rounds, coders gained familiarity with videos and determined the validity of the initial set of codes. Discrepancies in coding were discussed and resolved as a group. Furthermore, inductively, we added a new preliminary code whenever a video featured an experience of the MIB that could not be suitably coded into any existing categories. Bonding experience codes that frequently recurred were made into permanent codes. We reached data saturation finding repetition of codes during our coding of our final random sample of 159 videos. Code definitions are provided in Table S1 in Multimedia Appendix 1 with codes derived from the literature cited. Videos were coded under multiple codes when applicable. Interrater reliability (IRR) was acceptable (Krippendorff ɑ=0.67‐0.71) [29] in pairwise ratings of coders for coding 30 randomly selected videos. We used Excel (Microsoft Corp) to facilitate coding.

Our data abstraction from codes to categories was guided by the nature of the data and our research objectives [30]. Given the short-form nature of many TikTok videos with limited depth and our desire to closely represent the postpartum depressed mothers’ bonding experiences, we abstracted from codes to a higher-order category grouping without much interpretation. Thus, we chose not to use a thematic framework to avoid imposing external structures on the data. Instead, we used Miro boards to independently sort and group codes into categories based on similarities and relationships between codes. Categories had to be internally homogeneous and distinguishable from other categories. We then compared independently created categories among coders and aligned them to finalize the categories.

We reviewed creators’ profiles and videos to determine their country of residence and the approximate age of their child when the mothers experienced postpartum depression. To estimate the child’s age, we primarily used the age of the child when the video was created. However, if the mothers specifically mentioned experiencing depression during a particular period, such as the “first few months,” we used that age instead.

### Statistical Analysis

Because engagement metrics were skewed, we fit a negative binomial regression. Our dependent variables included likes, views, shares, and comment counts. The independent variables were the categories of video content. We also included the content creator’s number of followers (log-scaled to account for overdispersion) as an explanatory variable because it is generally associated with video engagement. We included all independent variables simultaneously (ie, no stepwise regression) in the model. We used Python (version 3.8.8; Python Software Foundation) for data analysis. Statistical tests were 2-sided with significance set at *P*<.05.

## Results

**Figure 1.**
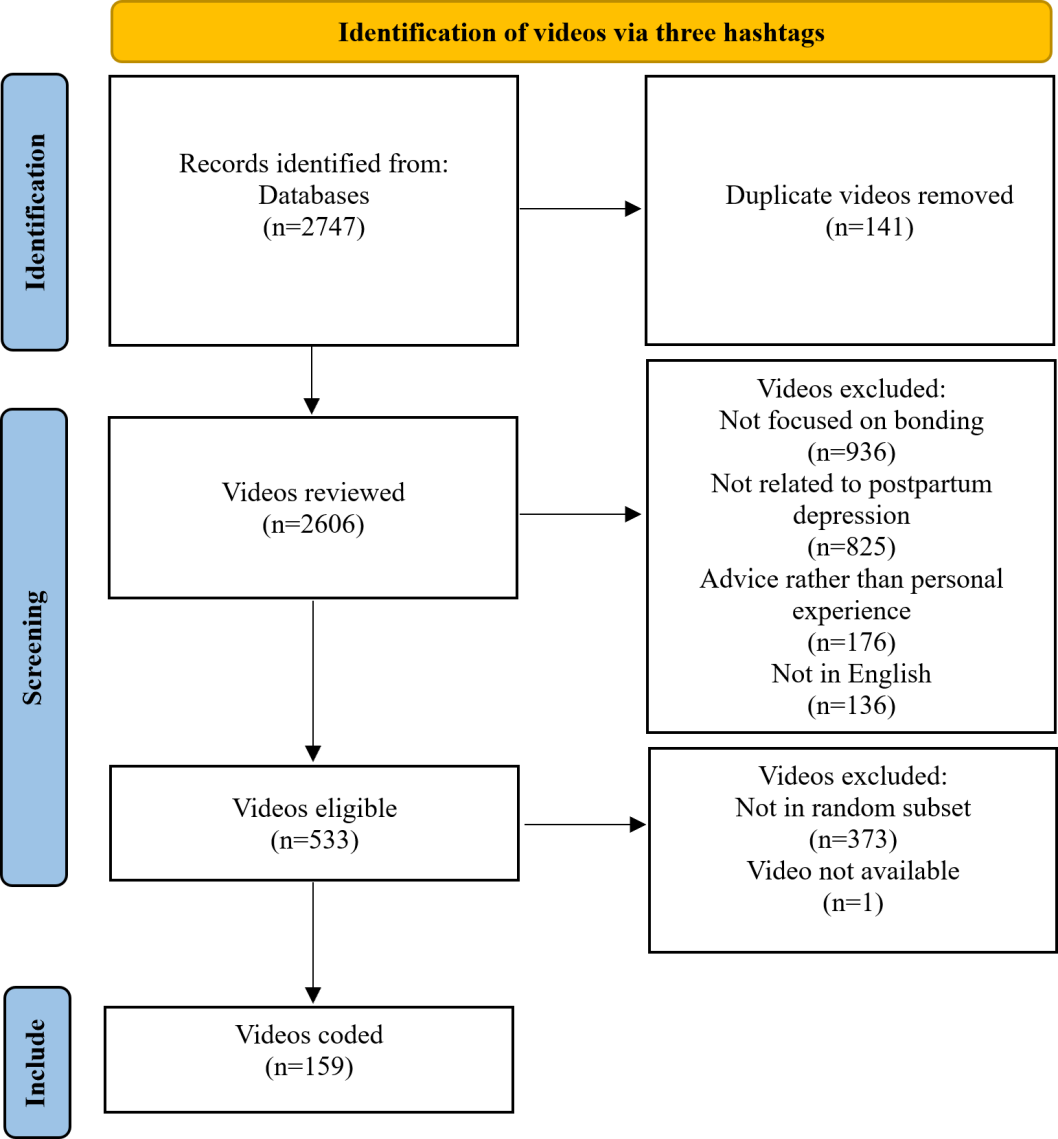
Flow diagram of included videos.

Figure 1 outlines the review process that resulted in 533 relevant videos.

### Characteristics of the Included Short-Form Videos

The 159 videos from 147 content creators in our analytic sample had a median of 42,200 (IQR 10,800‐15,6750) views, 4347 (IQR 970-12,100) likes, 35 (IQR 5-196) shares, and 80 (IQR 26-365) comments. Altogether, these videos have been viewed over 35 million times. Video creators (n=147) had a median of 31,800 (IQR 5265‐137,900) followers ranging from 46 to over 1,700,000. Creators were primarily based in the United States (141/159, 88%). Other countries represented included the United Kingdom (7/159, 4%), Canada (5/159, 3%), Australia (2/159, 1%), South Africa (1/159, 1%), the Philippines (1/159, 1%), and New Zealand (1/159, 1%). The country of one creator could not be determined (1/159, 1%). Most mothers experienced postpartum depression during the first year of their child’s life (147/159, 82%). Mothers of toddlers, most of whom had children younger than 2 years of age, were also represented (17/147, 12%). In some cases, the age of the children could not be determined (9/147, 6%).

### Content Categories

#### Overview

We abstracted 6 categories from the video codes, which are described below. Table 1 lists categories with their respective codes and examples.

**Table 1.** Categories with code examples of the content of depressed mothers’ bond with their child.

Category, code	Example video description
Navigating anxiety and anger	
Obsessive thoughts or worries about the child’s health or safety and compulsive behaviors to cope with it	The video shows a mother enacting an internal argument with her brain regarding her child’s safety when a family member holds the baby. Her brain worries that another family member will drop her baby and wants to take the baby back. Despite the creator trying to rationalize that the baby is safe in an effort to quell her intrusive thoughts, she feels a panic attack beginning.
Obsessive thoughts or worries of harming the baby and compulsive behaviors to cope with it	The creator was visibly upset as she shared ego-dystonic intrusive thoughts of harming her baby in the first few months postpartum. These included thoughts of dropping her baby. She could not control these thoughts, resulting in her having panic attacks and needing another person to stay with her. She reports being afraid to tell people, including her husband, but eventually, she did open up to her obstetrician.
Feelings of anger or aggression toward the child	The video shows a mother holding her head as she seems to reminisce. Text overlaid on the video describes a memory of wanting to throw her baby across the room when rocking the baby to sleep. But instead, she threw a piece of exercise equipment. Mother mouths “crazy” along with the lyrics from a song playing.
Involuntary emotional or mental responses	Video of mother verbally describing her postpartum depression relapse. She reports crying whenever she sees her child for no apparent reason. She starts tearing up after changing the camera angle from herself to show her baby.
Creating physical and emotional boundaries	
Mothers put up physical or emotional boundaries for fear of harming the baby	The creator stares at the camera as it zooms in with text overlaid on the video. Text relays an instance when she was in distress, waiting in the closet for a relative to come so she could avoid hitting her child. Music plays in the background.
Mothers put up physical or emotional boundaries for relief	The creator states that it is okay for moms to want to hide and get a break. Music in the background has lyrics about needing “me time” and “free time.”
Mothers try to minimize their child’s exposure to their distress or hope it does not or did not cause distress	The video shows a mother who appears to be putting the final touches on her appearance with text that she puts on a happy face for her baby despite going through postpartum depression and wanting to “curl in a ball and sleep.”
Overstimulated by too much sensory input from the child	The mother mentions that hearing her child cry causes her physical pain and agony.
Escaping from the caregiving role	Mother is tearful with overlaid text that she could never understand how people could leave their children behind until she had postpartum depression.
Overwhelmed by demands of caregiving	
Overwhelmed by caregiving responsibilities	The video shows a mother initially smiling but then gazing into the distance, thinking about something deeply. As she thinks, text bubbles appear and pile up on one another. These thoughts state things like: “What if she falls?”; “laundry”; “My house is a mess”; and “I shouldn’t have yelled.” Among the many thoughts piling up are: “I can’t keep this up” and “Am I a good mom?”
Depression makes it difficult for the mother to take care of their child	The video shows a mother’s internal struggle with her feelings of depression and lack of motivation to get off the couch to care for her child, as well as feelings of guilt about being an incompetent mother. Mother shares the thoughts racing in her mind, such as: “You’re a bad mom for not playing with him.”
Breastfeeding as a burdensome and distressing experience	The video shows a mother with a can of formula with the text: “I’m not okay. I bought a can of formula last night because I’m so burnt out over nursing.” Mother covers her face with her hands near the end of the video as if exhausted or ashamed with text that she “feel[s] like a failure for quitting.”
Not feeling the expected emotional connection or enjoyment with the child	The mother uses sound bite, saying, “This is how it feels?!” to express her surprise after finally feeling a strong connection to her child after struggling with postpartum depression (written in text).
Subverted expectations	
Feelings of guilt or regret regarding their care or relationship with their child	The video shows a montage of videos of the creator’s child as a newborn. In the video text, she expresses regret that she could not cherish her child’s newborn phase because she was overtired and overwhelmed.
Feel incompetent as a mother	The video shows a mother on one side of a door embracing her child with text that she is trying to be a good mom. On the other side of the door, she personifies postpartum depression, trying to break through the door to disrupt the mother’s efforts.
Enduring and finding strength through the challenge of postpartum depression	
Mother finds hope or resilience through their relationship with their child	The video shows a collage of photos of a mother and baby appearing happy. The text says: “Postpartum was not good to me, but she is the sole reason I’m still here and always will be.”
Responsibility or duty to the child	The video has narration stating difficulty getting out of bed but having to do so “to keep them [children] alive.” The video goes on to say that although the mother feels like she is “hanging on by a thread,” she continues to get up each day for her children.
Positive feelings or thoughts toward the child or a positive relationship with the child despite postpartum depression	The mother poses a question to herself, asking if she wants to see life with her baby even at the cost of struggling with postpartum depression and thoughts of suicide. After she answers yes, a montage of photos and videos of happy moments of the mother and baby together begins.
Can’t remember early life	
Postpartum depression makes it hard to remember child’s early life	The video shows a mother pretending to pull a knife out of her chest with text that she could not remember the newborn stage of her child’s life because she was “so depressed.” In the video description, the creator writes that she wishes she had taken more videos and pictures during that time.

#### Navigating Anxiety and Anger

Mothers with depression shared feelings of anxiety and anger when interacting with their children. Many mothers experienced perseverative intrusive thoughts of something terrible happening to their child. These thoughts frequently related to the child passing away while asleep, though scenarios varied from the child choking while eating to others making the child sick. One creator mouthed a sound bite to recreate a conversation with her obstetrician, who instructed her that pulling over her car every time her baby fell asleep to check if her baby was breathing was a sign of postpartum anxiety. Some mothers had ego-dystonic intrusive thoughts of harming their baby, but more often, anger toward the child was a genuine feeling. Triggers for anger included difficulties with child sleep, crying, screaming, and sensory overstimulation. Still, more broadly, many mothers noted that their thoughts and feelings arose without warning. Only a few mothers explicitly mentioned anxiety or anger as a manifestation of postpartum depression. For example, one mother said, “It can come in so many different forms: anger, rage, depression, sadness.”

In reaction to anxiety and anger, mothers engaged in behaviors to protect their children. For example, mothers compulsively checked if their child was safe while sleeping or vigilantly watched them, often resulting in poor sleep; some used baby monitoring devices to try to alleviate their fears. Mothers sometimes displaced their anger by hitting objects or themselves. In addition, they physically separated themselves from their child if they had these thoughts of hurting them. Several mothers brought up reluctance to share these feelings with their doctors and family members for fear of negative judgment.

#### Creating Physical and Emotional Boundaries

Mothers exhibited avoidant behaviors or attitudes toward their children. Some mothers expressed the need for a break from caregiving for personal relief due to childcare-related stress. Sensory overload, including loud noises and frequent touch, was a common stressor. One mother described feeling physically in pain due to hearing their child’s cries. Other mothers needed to physically relocate in order to calm their fluctuating emotions (sadness, anger, and frustration). For example, some women who felt frustrated would remove themselves from the vicinity of their child to avert lashing out at the child. In addition to personal relief, women expressed fear that their emotional state may negatively impact their child. For example, some creators disclosed that they would conceal their own distress by leaving the room when crying or putting on a happy façade in hopes of minimizing the impact their observable distress may have on their child. Other women described having frequent fantasies where they are forced out of a caregiving role (eg, sickness, suicidal thoughts, and running away).

#### Overwhelmed by Demands of Caregiving

Mothers struggling with postpartum depression were often overwhelmed by daily caregiving tasks, causing them to feel depleted or on the verge of a breakdown. As the tasks piled up on each other, many shared a feeling of inadequacy, doubting their competence as mothers. These tasks include feeding, diapering, meal preparation, toileting, and managing sleep routines. Challenges with breastfeeding were pronounced, leaving mothers feeling incapable, which further worsened their mental health. Consequently, some moms switched to formula feeding, though they felt guilty about whether this was best for their child. Caregiving was often described as all-consuming, limiting mothers’ time to care for themselves, as evidenced by several videos showing mothers alone in a secluded area (eg, bathroom) away from their children. Relatedly, many women noted a lack of caregiving support, and several videos featured women caring for multiple children. Depressive symptoms such as low energy made it hard to care for their child as desired. Some women were able to get support from family at times when depression left them unable to take care of their children.

#### Subverted Expectations

The gap between the idealized image of motherhood and experiencing motherhood with postpartum depression left women with negative images of themselves as a mother. Due to depression, mothers expressed being unable to experience the joy and bond with their children that they anticipated. Consequently, mothers expressed feeling incompetent, with many creators asking, “Am I a good mother?” Mothers revealed feeling shame and guilt over their perceived nonconventional emotional state. One mother recounted expecting an instantaneous bond with her child and described feeling shame and guilt when she did not feel the connection early on. Depression also caused tiredness and sensitive emotional states, making it difficult for mothers to fulfill their own image of an ideal parent. Subverted expectations affected mothers both in the present and past. Some mothers expressed regret over missing out on the regular interactions they believed a parent and child should experience in early life due to depression or related tiredness, feeling overwhelmed, or delayed bonding.

#### Enduring and Finding Strength Through the Challenge of Postpartum Depression

Mothers with postpartum depression shared how they found the strength to persevere through their relationship with their children. Despite struggling to have the energy to get up each day and fulfill the demanding tasks of motherhood due to their depression, these mothers identified that their children were their primary motivating factor to carry on. Often, mothers stated that the smiles on their children’s faces were uplifting. Music used in these videos often had resilience-themed lyrics such as “Rise up,” “You are my sunshine,” and “Don’t give up.” Some mothers shared a neutral sentiment, feeling that they must take care of their child—and would not let depression hinder their caregiving. Additionally, some mothers expressed their children sparked their desire to seek treatment.

#### Can’t Remember Early Life

Some mothers noticed they had little memory of their child’s early life months. These missing memories include important milestone moments, as well as day-to-day caregiving. With this realization came deep sadness that postpartum depression had robbed these special moments from them. This realization often came when their child was older or as their depression began to improve. Frequently, mothers were only reminded of these memories when presented with a picture of their child. Some women used photo montages in their videos to symbolize missed memories.

### User Engagement

User engagement statistics for these categories across all videos are provided in Table 2. Overall, videos featuring content on navigating anxiety and anger, as well as subverted expectations, received the most views, likes, and comments. Users most frequently shared videos on subverted expectations and creating physical and emotional boundaries. Sensitivity analyses using normalized (log-scaled) follower counts found similar trends (Table S2 in Multimedia Appendix 1).

**Table 2.** User engagement of videos by content category.

Category	Videos, n (%)^a^	Views, median (IQR)	Likes, median (IQR)	Shares, median (IQR)	Comments, median (IQR)
Overwhelmed by demands of caregiving	64 (40)	39,700 (10,586‐142,300)	3966 (1047‐14,900)	52 (7‐282)	128 (42‐550)
Subverted expectations	56 (35)	50,050 (17,575‐171,275)	4419 (1191‐17,225)	80 (6‐292)	147 (31‐375)
Navigating anxiety and anger	53 (33)	67,600 (18,800-155,800)	6123 (1654‐13,600)	49 (5‐290)	132 (40‐532)
Enduring and finding strength through the challenge of postpartum depression	26 (16)	27,600 (11,025-86,925)	1856 (553‐10,734)	10 (1‐110)	40 (16‐285)
Creating physical and emotional boundaries	25 (16)	42,200 (10,800-91,800)	2774 (867‐9932)	69 (4‐223)	77 (25‐315)
Can’t remember early life	10 (6)	32,450 (25,125-211,075)	4180 (1970‐6808)	24 (10‐89)	36 (17‐97)

a Data are expressed as n (%) of 159 videos because a single video can have multiple categories.

In regression analyses, videos on navigating anxiety and anger and subverted expectations increased the views, likes, shares, and comments by 61% to nearly 400% (Table 3). Content featuring the overwhelmed by demands of caregiving category also increased likes, shares, and comments, but not views. Content on resilience in the face of postpartum depression was more highly liked (rate ratio 1.60, 95%CI 1.00‐2.57) but less likely to receive comments (rate ratio 0.48, 95%CI 0.30‐0.78). Across all metrics, users engaged less with videos on creating physical and emotional boundaries. We found a small negative association between the log number of followers and all engagement types. Sensitivity analysis of creators with less than the median number of followers mostly corroborated these main findings with the following exceptions: the associations of enduring and finding strength through the challenge of postpartum depression, overwhelmed by demands of caregiving categories, and number of followers with engagement were consistently negative, nonsignificant, and positive, respectively (Table S3 in Multimedia Appendix 1).

**Table 3.** Association between video content categories and user engagement.

Variable	Risk ratio (95% CI)	*P* value
Views		
Overwhelmed by demands of caregiving	1.12 (0.78‐1.61)	.53
Subverted expectations	1.72 (1.22‐2.43)	.002^a^
Navigating anxiety and anger	1.61 (1.09‐2.38)	.02^a^
Enduring and finding strength through the challenge of postpartum depression	0.75 (0.46‐1.20)	.23
Creating physical and emotional boundaries	0.40 (0.26‐0.61)	*<*.001^a^
Can’t remember early life	0.95 (0.47‐1.92)	.88
Creator’s number of followers^b^	0.92 (0.85‐0.98)	.02^a^
Likes		
Overwhelmed by demands of caregiving	1.61 (1.13‐2.31)	.009^a^
Subverted expectations	3.61 (2.55‐5.11)	*<*.001^a^
Navigating anxiety and anger	3.96 (2.69‐5.85)	*<*.001^a^
Enduring and finding strength through the challenge of postpartum depression	1.60 (1.00‐2.57)	.052
Creating physical and emotional boundaries	0.30 (0.19‐0.46)	*<*.001^a^
Can’t remember early life	1.45 (0.72‐2.94)	.30
Creator’s number of followers^b^	0.89 (0.83‐0.96)	.002^a^
Shares		
Overwhelmed by demands of caregiving	1.78 (1.24‐2.55)	.002^a^
Subverted expectations	2.95 (2.09‐4.18)	*<*.001^a^
Navigating anxiety and anger	2.45 (1.66‐3.61)	*<*.001^a^
Enduring and finding strength through the challenge of postpartum depression	1.09 (0.68‐1.76)	.72
Creating physical and emotional boundaries	0.63 (0.41‐0.98)	.04^a^
Can’t remember early life	0.87 (0.43‐1.77)	.70
Creator’s number of followers^b^	0.89 (0.83‐0.95)	.001^a^
Comments		
Overwhelmed by demands of caregiving	2.67 (1.87‐3.83)	*<*.001^a^
Subverted expectations	2.78 (1.97‐3.94)	*<*.001^a^
Navigating anxiety and anger	1.89 (1.28‐2.79)	.001^a^
Enduring and finding strength through the challenge of postpartum depression	0.48 (0.30‐0.78)	.003^a^
Creating physical and emotional boundaries	0.44 (0.29‐0.68)	*<*.001^a^
Can’t remember early life	0.51 (0.25‐1.05)	.07
Creator’s number of followers^b^	1.00 (0.93‐1.07)	.99

a Values are significant.

b Number of followers is in log scale.

## Discussion

### Principal Findings

This qualitative content analysis is the first study to examine the MIB in the context of postpartum depression using short-form videos. Nearly 150 mothers shared their experiences bonding with their children, resulting in 6 categories that advance understanding of this topic. Analysis of engagement metrics across categories added information on topics that resonated most with users. These findings support the use of short-form videos to enhance our understanding of lived experiences.

### Video Content

Many of these categories reflect themes in the literature. The subverted expectations category aligns with prior studies discussing the clash between mothers’ personal or societal expectations and the lived reality of postpartum depression [31,32]. Regarding bonding specifically, we found women worried that they were not the “good mother” they expected and felt associated guilt and shame. Unrealistic expectations of having an instantaneous emotional connection with their child contributed frequently to their mothering assessment. Regret was another prominent feeling, with mothers reporting remorse for missing out on moments with their children. As in Beck’s [12] paper, depression was frequently described as “robbing” moms of precious time with their young children.

Several studies report that mothers with depression feel overwhelmed by caregiving responsibilities. Our results similarly find that the symptoms of depression, such as lack of energy, low mood, and lack of joy, compound the everyday challenges of raising a young child [12,33]. We found that poor maternal sleep and breastfeeding often contributed to mothers feeling like they could not meet these demands. Mothers could not refuel because they often could not take a break, or as one mother described, get “me” time.

Although anxiety and anger have long been recognized as manifestations of postpartum depression [34,35], few women in this study directly associated their anxiety or anger with their depression. Previous qualitative and quantitative studies of postpartum depression [35,36] describe symptoms such as intense intrusive thoughts and outbursts of anger, both provoked and unprovoked, which align with the experiences categorized as navigating anxiety and anger in this study. The low attribution of these symptoms to depression in this study might result from greater awareness and screening for postpartum depression compared to anxiety or anger. Therefore, even if anxiety or anger are sequelae or independent conditions, mothers might not have perceived them as symptoms of depression.

Despite this, mothers commonly described anxiety and anger as negatively affecting their bond with their children. Experiences of obsessions and compulsions, as highlighted in other qualitative studies [12] and our research, demonstrate the significant role that postpartum-specific anxieties have on the MIB. Indeed, postpartum anxiety impairs bonding, leading to pathological anger and infant-focused anxiety [37]. Prior research [12,36] has also shown that depressed mothers experience anger but find ways to avoid taking it out on their children, akin to how mothers on TikTok coped.

The creating physical and emotional boundaries category, characterized by behaviors or wishes to be separated from the infant, shared some similarities with a study of posts on a postpartum depression internet forum [17]. That study found that desires to be separated from the child manifested with thoughts or feelings of hurting the child. However, mothers in this study were more avoidant rather than aggressive. In addition, though some mothers noted thoughts of suicide, descriptions were less explicitly expressed in videos than in internet forum posts. These differences may be due to reluctance to share these thoughts by video, which is less identity-preserving.

In contrast, the enduring and finding strength through the challenge of postpartum depression and can’t remember early life categories are less represented in the literature. Prior studies similarly found that mothers with postpartum depression expressed positive feelings toward their child or felt an obligation to meet their child’s needs despite postpartum depression. Our results go a step further: for some mothers, their bond with their child offered comfort and reassurance. We know of a similar finding in one study of West African mothers residing in the United Kingdom who described their baby as a source of pride or distraction from distress [38]. Some mothers had difficulty remembering their child’s early life because they were trying to “survive.” Without these memories that connect and strengthen the bond between mother and child, these mothers felt deep sadness and regret. Traumatic events, such as childbirth trauma, can cloud memory, but only 2 included videos in this category mentioned trauma. Given the lack of prior literature on this phenomenon, it should be further explored in future work.

Determining mothers’ motivations for sharing these personal experiences on social media is challenging without direct inquiry. Studies have shown that self-expression is a primary motivation for people with mental health conditions to share content on social media [39-41]. Creators, particularly on platforms like TikTok, are often willing to share personal experiences because they perceive a safe environment free from negative judgment [39,40]. This perceived safety encourages those with stigmatized conditions or identities to share their experiences [39,42]. Other motivations, such as the desire to receive social support or gain social status, are possible and could influence the type of content shared. The congruence of our findings with previous research using interviews, including in women diagnosed with clinical depression [12], lends credibility to our approach of analyzing short-form videos. Future research should interview creators to better understand their motivations and how these motivations influence the content they produce.

The technological features of social media platforms likely influence how women express their lived experiences. Women used the various multimedia affordances provided by the video-sharing platform to share their experiences. In addition to text, they often added sound bites or music to convey meaning. Visual effects such as photo montages and re-enactments of how postpartum depression affected bonding were another way mothers used the medium. Although most videos were less than 30 seconds, this multimodal content added to the richness of the content.

### User Engagement

Videos featuring 3 categories well-represented in the literature—subverted expectations, navigating anxiety and anger, and overwhelmed by caregiving—received more views, likes, shares, and comments. According to the uses and gratifications theory [43], people engage with content that meets their desires and needs. Users “like” content to express appreciation for the material they find relevant, interesting, or emotionally engaging [39,44]. Users are more likely to engage with mental health content that shares personal experiences and experiential knowledge [45,46]. This trend is noticeable on TikTok, where users frequently share personal stories and information, often presented in creative or humorous ways. In addition, users are drawn to experiences that resonate with their own [39,47]. The challenges of motherhood, many of which are unexpected, are relatable experiences represented by these categories. However, in postpartum depression, these challenging experiences pervade the mother’s thoughts and actions, impairing the bond with their child.

Decreased likes, shares, and comments for the creating physical and emotional boundaries category indicate that users were less inclined to engage with videos about taking a break or fantasizing about separation from their child, possibly due to the stigma associated with appearing to want to abandon their responsibilities as a mother. However, content featuring anger generated more engagement, suggesting that anger without the expectation of abandoning the mother role may be the key difference. Content on resilience was well-received, nearing significance for increased likes. However, these videos are less likely to be commented on. This may reflect that users wanted to show support but may not have personally felt resilient or that it was actionable (eg, sharing advice). A study of depression on the Chinese version of TikTok found parallel results where positive portrayals of general depression had less engagement, which was attributed to positive content garnering less sympathy [48].

Our findings are further strengthened by the observation that videos from creators with small or modest followings were well-represented and received significant engagement. This suggests that diverse perspectives on postpartum depression, not just those from influencers with large followings, are valued. This could be attributed to TikTok’s algorithm, which promotes videos that people consistently watch and interact with to a broader audience, resulting in increased engagement. The potential of content creators with small followings to create a viral video is one of the draws for people to create content on TikTok [49].

### Clinical Implications

Our findings provide practical ways for clinicians to support bonding in depressed mothers. Across all these categories, mothers with postpartum depression tried various ways to cope with the challenges of bonding with their children. However, internalization of their bonding difficulties caused self-blame with associated shame and guilt, as well as isolation. Normalizing mothers’ experiences, given their commonality and the resulting shame, is therapeutic. For instance, clinicians can reassure mothers that it often takes time to develop an emotional bond with their child. Based on the high engagement, these videos may already be beneficial by normalizing these experiences. Self-compassion approaches that reduce shame and guilt effectively enhance bonding and reduce depression [50,51]. Sharing ways to improve sleep for moms and infants and strengthen social support would be helpful for overwhelmed mothers, especially those with multiple young children. Health care professionals can change the narrative by emphasizing that not every mother can breastfeed.

Further, our findings indicate that assessment of bonding in the context of postpartum depression should consider anxiety, anger, and parenting stress. Asking only about depressive symptoms will likely miss many women with bonding difficulties. Although clinicians may be reluctant to ask about anger toward the child or the mother’s escape fantasies, our results suggest that they should. It is important to acknowledge that mothers’ reluctance to share their struggles because of fears about child protective services removing their children is not without basis. Informing mothers on the difference between what is considered maltreatment and what is not may provide reassurance. We believe giving mothers information and allowing them to share as they so choose is a beneficial intervention within itself and opens the door to future conversations.

### Limitations

Although our findings are well supported by prior work, only publicly available videos with many views were reviewed. This enabled us to examine videos that resonate with many users, but further work is needed to determine if these categories are truly representative of bonding for women struggling with depression. In addition, while many social media researchers use hashtags to identify and learn about specific phenomena [22], it is unknown whether mothers in the videos have depressive symptoms or clinical depression. Nonetheless, subclinical depressive symptoms alone can negatively affect bonding [52]. In addition, demographic information such as maternal age, race, or sex could not be determined. We did not see videos featuring men with postpartum depression in our video corpus, highlighting an area for future investigation. The vast majority of women were from the United States followed by other Western countries. This is likely because we required videos to be in English. The concurrent findings in mothers from non-English speaking cultures are reassuring, but our approach limits generalizability.

Another limitation is the relatively low but acceptable IRR for the codes. The low IRR reflects the challenges of applying 21 nonmutually exclusive potential codes to short-form videos. Moreover, these codes were applied to 30 overlapping videos used for calculating IRR between each pair of coders, resulting in some of the codes being rarely applied. Consequently, even a single discrepancy between 2 coders when applying a code could lead to a moderate Krippendorff ɑ reliability score. Two of the 3 codes with the lowest IRR of 0 (“Mother perceives their child has negative feelings about them” and “Mothers put up physical or emotional boundaries for relief”) were derived from the prior literature review. This suggests that these 2 codes may have been less relevant to the current dataset. As a result, one of these codes was excluded when codes were abstracted into categories (“Mother perceives their child has negative feelings about them”). While coders conducted several rounds of review to determine if initial deductive codes were present, further rounds may have helped validate these codes and improve IRR.”

### Conclusions

Overall, our research demonstrates that short-form videos provide a meaningful lens through which to explore mothers’ experiences with postpartum depression, particularly its impact on the MIB. Knowledge gleaned helps us understand mothers’ specific challenges and thereby develop ways to support them.

## Supplementary material

10.2196/59125 Multimedia Appendix 1 Codes and definitions of content of depressed mothers’ bond with their child; normalized user engagement of videos by content category; association between video content categories and user engagement for creators with below the median number of followers; and references.

## Abbreviations

IRR: interrater reliability
MIB: maternal-infant bonding
RR: rate ratio
