# Supplementary material for: Postpartum Depression and Maternal-Infant Bonding Experiences in Social Media Videos: Qualitative Content Analysis
Source: JMIR Infodemiology. 2025 May 15;5:e59125. doi: 10.2196/59125 (PMC12122829; doi:10.2196/59125)
Supplement: Multimedia Appendix 1 [file infodemiology-v5-e59125-s001.docx]

**Table S1.** Codes and definitions of content of depressed mothers’ bond with their child

| **Code** | **Definition** |
| --- | --- |
| Obsessive thoughts or worries about the child's health or safety and compulsive behaviors to cope with it [1,2] | The mother mentions worrisome thoughts or engages in behaviors related to the safety of the child. For example, worries that the child is too thin or could stop breathing when falls asleep. Another example is frequently checking to make sure the child is okay. |
| Obsessive thoughts or worries of harming baby and compulsive behaviors to cope with it | The mother mentions worrisome thoughts or engages in behaviors related to harming the child. For example, mother has unwanted thoughts that she will harm the child. Another example would be the mom not holding the baby because she is afraid she will drop the child. |
| Feelings of anger or aggression towards the child^a^ [3,4] | The mother expresses thoughts or feelings of harming the child or has behaved in ways that are aggressive towards the child (verbal or physical, like yelling, hitting, etc.). |
| Involuntary emotional/mental responses | The mother has an unelicited or uncontrollable emotional/mental response towards or in reaction to the child. The mother may not be able to identify why they are having this response. |
| Mothers put up physical or emotional boundaries for fear of harming the baby^b^ [4,5] | The creator expresses physical or emotional separation from the child to avoid harming the child. For example, the mother avoids or separates from the child so they do not harm the child. |
| Mothers put up physical or emotional boundaries for relief^c^ [4,5] | The mother expresses physical or emotional separation from the child to alleviate distress. For example, the mother avoids the child to decrease stress or take a break. |
| Mothers try to minimize their child's exposure to their distress or hopes it does not/did not cause distress | The mother tries to avoid showing negative emotions when with the child or expresses a hope that prior instances did not have an adverse effect on the child. For example, the mother avoids crying in front of their child. |
| Overstimulated by too much sensory input from the child | The mother expresses feeling overwhelmed or distressed because of too much sensory input from the child. Senses are vision, sound, smell, touch, or taste. For example, low noises, being touched a lot, or strong smells |
| Escaping from the caregiving role | The mother expresses a desire to cease their relationship or caregiving role with their child. |
| Overwhelmed by caregiving responsibilities [2] | Mother expresses feeling burdened by taking care (e.g., diapering, putting the child to sleep, etc., except breastfeeding) of the child. For example, the mother identifies too many tasks to complete. |
| Depression makes it difficult for the mother to take care of their child [6] | The mother attributes difficulty in caregiving to symptoms of depression. For example, the mother feels too exhausted to take care of the child. |
| Breastfeeding as a burdensome and distressing experience [7] | The mother states that breastfeeding the infant is frustrating or burdensome. |
| Not feeling the expected emotional connection or enjoyment with the child ^†^ [8] | The mother does not feel like they have a close relationship with the child or feels like depression makes it hard for them to enjoy their child. |
| Feelings of guilt or regret regarding their care or relationship with their child [4,7] | The mother expresses thoughts or feelings of harming the child or has behaved in ways that are aggressive towards the child (verbal or physical, like yelling, hitting, etc.). |
| Feel incompetent as a mother [2] | The mother expresses a lack of confidence in their ability to take care of their child. For example, the creator states that she is not a good mother. |
| Mother find hope or resilience through their relationship with their child [2] | Mother expresses that their child helped/helps them while they were/are depressed. For example, the mother mentions that they persevered with postpartum depression because of their child. |
| Responsibility/Duty to the child | The mother expresses that the child is a responsibility/duty they have. For example, the mother states they still have to care for their child. |
| Positive feelings/thoughts towards the child or a positive relationship with the child despite postpartum depression | Mother expresses a positive regard for or relationship with the child. For example, the mother mentions loving their child. |
| Postpartum depression makes it hard to remember child's early life | The mother notes difficulty remembering the child's early life. For example, the mother states not remembering the first few years of the child's life. |
| Mother perceives their child has negative feelings about them [6] | The mother expresses that the child feels negatively about them. For example, the mother thinks that their child does not like them. |
| Going through the motions with caregiving [1,4] | The mother's provides care by attending solely to the child's basic needs or in a mechanical fashion. |

Note: Codes derived from the literature are cited. For modified codes, we list the original code/categories/themes derived from the literature.

^a^Original code: Feelings of anger towards the child

^b^Original code: Mothers put up physical or emotional boundaries for relief or for fear of harming the baby

^c^Original code: Not feeling an emotional connection with the child

**Table S2.** Normalized user engagement of videos by content category

| Category | Videos, n (%)^a^ | Views, median (IQR) | Likes, median (IQR) | Shares, median (IQR) | Comments, median (IQR) |
| --- | --- | --- | --- | --- | --- |
| Overwhelmed by demands of caregiving | 64 (40) | 3618 (1181-14,383) | 370 (116-1478) | 7 (1-26) | 14 (4-55) |
| Subverted expectations | 56 (35) | 4907 (1683-12,569) | 432 (103-1471) | 6 (1-26) | 13 (3-34) |
| Navigating anxiety and anger | 53 (33) | 6074 (2,094-15,822) | 455 (205-1108) | 4 (1-24) | 12 (5-52) |
| Enduring and finding strength through the challenge of postpartum depression | 26 (16) | 2885 (1372-6316) | 233 (54-1065) | 1 (0-11) | 6 (2-28) |
| Creating physical and emotional boundaries | 25 (16) | 3377 (1324-12,498) | 272 (85-945) | 5 (1-21) | 7 (4-31) |
| Can’t remember early life | 10 (6) | 2851 (2190-23,353) | 354 (171-740) | 2 (1-10) | 3 (2-10) |

^a^Data are expressed as No. (%) of 159 online videos because a single video can have multiple categories.

**Table S3**. Association between video content categories and user engagement for creators with below the median number of followers

| Variable | Risk ratio (95% CI) | *P* value |
| --- | --- | --- |
| **Views** |  |  |
|  |  |  |
| Overwhelmed by demands of caregiving | 0.73 (0.44-1.20) | .211 |
| Subverted expectations | 1.61 (0.96-2.69) | .070 |
| Navigating anxiety and anger | 1.79 (1.06-3.01) | *.029* |
| Enduring and finding strength through the challenge of postpartum depression | 0.18 (0.09-0.34) | *<.001* |
| Creator’s number of followers^a^ | 1.60 (1.37-1.87) | *<.001* |
| **Likes** |  |  |
|  |  |  |
| Overwhelmed by demands of caregiving | 0.72 (0.44-1.19) | .203 |
| Subverted expectations | 2.60 (1.55-4.34) | *<.001* |
| Navigating anxiety and anger | 3.41 (2.02-5.75) | *<.001* |
| Enduring and finding strength through the challenge of postpartum depression | 0.30 (0.15-0.58) | *<.001* |
| Creator’s number of followers^a^ | 1.76 (1.51-2.06) | *<.001* |
| **Shares** |  |  |
|  |  |  |
| Overwhelmed by demands of caregiving | 1.07 (0.65-1.77) | .792 |
| Subverted expectations | 3.12 (1.87-5.22) | *<.001* |
| Navigating anxiety and anger | 1.18 (0.70-1.99) | .545 |
| Enduring and finding strength through the challenge of postpartum depression | 0.41 (0.21-0.79) | *.008* |
| Creator’s number of followers^a^ | 1.87 (1.59-2.19) | *<.001* |
| **Comments** |  |  |
|  |  |  |
| Overwhelmed by demands of caregiving | 2.13 (1.29-3.52) | *.003* |
| Subverted expectations | 3.10 (1.86-5.19) | *<.001* |
| Navigating anxiety and anger | 1.31 (0.78-2.22) | .305 |
| Enduring and finding strength through the challenge of postpartum depression | 0.37 (0.19-0.73) | *.004* |
| Creator’s number of followers^a^ | 1.62 (1.39-1.90) | *<.001* |

^a^Number of followers is in log scale.

^b^Italicized values are significant.

^c^The following categories were excluded from regression analysis due to low counts: Creating physical and emotional boundaries (n = 6), Can't remember early life (n = 4)

**References**

1. Knudson‐Martin C, Silverstein R. Suffering in Silence: A Qualitative Meta‐Data‐Analysis of Postpartum Depression. J Marital Fam Ther 2009 Apr;35(2):145–158. doi: 10.1111/j.1752-0606.2009.00112.x

2. Holopainen A, Hakulinen T. New parents’ experiences of postpartum depression: a systematic review of qualitative evidence. JBI Database Syst Rev Implement Rep 2019 Sep;17(9):1731–1769. doi: 10.11124/JBISRIR-2017-003909

3. Johansson M, Benderix Y, Svensson I. Mothers’ and fathers’ lived experiences of postpartum depression and parental stress after childbirth: a qualitative study. Int J Qual Stud Health Well-Being 2020 Jan 1;15(1):1722564. doi: 10.1080/17482631.2020.1722564

4. Beck CT. Postpartum Depressed Mothers’ Experiences Interacting With Their Children. Nurs Res 1996;45(2):98–104. doi: 10.1097/00006199-199603000-00008

5. Beck CT. Postpartum Depression: A Metasynthesis. Qual Health Res 2002 Apr;12(4):453–472. doi: 10.1177/104973202129120016

6. Nilav MJ. Nature and Impact of Postpartum Depression on Women’s Life. University of Dhaka; 2023.

7. Gashlin LA. Beyond postpartum depression: A qualitative phenomenological examination into the evolving mother-child relationship. Chestnut Hill College; 2014.

8. Adlington K, Vasquez C, Pearce E, Wilson CA, Nowland R, Taylor BL, et al. ‘Just snap out of it’ – the experience of loneliness in women with perinatal depression: a Meta-synthesis of qualitative studies. BMC Psychiatry 2023 Feb 28;23(1):110. doi: 10.1186/s12888-023-04532-2
